# Topology-based sparsification of graph annotations

**DOI:** 10.1093/bioinformatics/btab330

**Published:** 2021-07-12

**Authors:** Daniel Danciu, Mikhail Karasikov, Harun Mustafa, André Kahles, Gunnar Rätsch

**Affiliations:** Department of Computer Science, Biomedical Informatics Group, ETH Zurich, Zurich, Switzerland; Biomedical Informatics Research, University Hospital Zurich, Zurich, Switzerland; Department of Computer Science, Biomedical Informatics Group, ETH Zurich, Zurich, Switzerland; Biomedical Informatics Research, University Hospital Zurich, Zurich, Switzerland; Swiss Institute of Bioinformatics, Zurich, Switzerland; Department of Computer Science, Biomedical Informatics Group, ETH Zurich, Zurich, Switzerland; Biomedical Informatics Research, University Hospital Zurich, Zurich, Switzerland; Swiss Institute of Bioinformatics, Zurich, Switzerland; Department of Computer Science, Biomedical Informatics Group, ETH Zurich, Zurich, Switzerland; Biomedical Informatics Research, University Hospital Zurich, Zurich, Switzerland; Swiss Institute of Bioinformatics, Zurich, Switzerland; Department of Computer Science, Biomedical Informatics Group, ETH Zurich, Zurich, Switzerland; Biomedical Informatics Research, University Hospital Zurich, Zurich, Switzerland; Swiss Institute of Bioinformatics, Zurich, Switzerland; Department of Biology, ETH Zurich, Zurich, Switzerland

## Abstract

**Motivation:**

Since the amount of published biological sequencing data is growing exponentially, efficient methods for storing and indexing this data are more needed than ever to truly benefit from this invaluable resource for biomedical research. Labeled de Bruijn graphs are a frequently-used approach for representing large sets of sequencing data. While significant progress has been made to succinctly represent the graph itself, efficient methods for storing labels on such graphs are still rapidly evolving.

**Results:**

In this article, we present RowDiff, a new technique for compacting graph labels by leveraging expected similarities in annotations of vertices adjacent in the graph. RowDiff can be constructed in linear time relative to the number of vertices and labels in the graph, and in space proportional to the graph size. In addition, construction can be efficiently parallelized and distributed, making the technique applicable to graphs with trillions of nodes. RowDiff can be viewed as an intermediary sparsification step of the original annotation matrix and can thus naturally be combined with existing generic schemes for compressed binary matrices. Experiments on 10 000 RNA-seq datasets show that RowDiff combined with multi-BRWT results in a 30% reduction in annotation footprint over Mantis-MST, the previously known most compact annotation representation. Experiments on the sparser Fungi subset of the RefSeq collection show that applying RowDiff sparsification reduces the size of individual annotation columns stored as compressed bit vectors by an average factor of 42. When combining RowDiff with a multi-BRWT representation, the resulting annotation is 26 times smaller than Mantis-MST.

**Availability and implementation:**

RowDiff is implemented in C++ within the MetaGraph framework. The source code and the data used in the experiments are publicly available at https://github.com/ratschlab/row_diff.

## 1 Introduction

The exponential increase in global sequencing capacity (Stephens *et al.*, 2015) and the resulting growth of public sequence repositories have created an urgent need for the development of compact representation schemes of biological sequences. Such schemes should not only maintain all relevant biological sequence variation but also provide fast access for sequence search and extraction. After initial attempts focused on the lossless compression of full sequences, e.g. using the Burrows–Wheeler transform (Cox *et al.*, 2012), the field soon turned toward representing a proxy of the input sequences instead: the sets of all *k*-mers contained in them. For this, any recurrent occurrence of a substring of length *k* in the input is represented by a unique *k*-mer, forming a *k*-mer set. A query of a given sequence against the input text can then be replaced by exact *k*-mer matching against the set. Longer strings are queried as a succession of *k*-mers. Although it is a loss representation of the input (as, e.g. repeats longer than *k* are collapsed), constructing *k*-mer sets has proved highly useful in practice (Bradley *et al.*, 2019; Breitwieser *et al.*, 2018; Karasikov *et al.*, 2020a; Ondov *et al.*, 2016).

### 1.1 Representation of *k*-mer sets

Various representations have been developed to balance the trade-off between the space taken by the *k*-mer set and query time or representation accuracy. Conceptually, the *k*-mer set fully defines a vertex-centric de Bruijn graph, where each *k*-mer forms a vertex and arcs are represented implicitly, based on whether any two vertices share a *k—*1 overlap. The simplest representations are bitmaps or (perfect) hash-tables that indicate the presence or absence of any possible *k*-mer over the input alphabet in the input text. While non-optimal in space, they offer constant-time query of *k*-mers. More compact representations use approximate membership query data structures to probabilistically represent a de Bruijn graph (Benoit *et al.*, 2015; Chikhi and Rizk, 2013) or utilize succinct de Bruijn graphs (a generalization of the Burrows–Wheeler transform) (Bowe *et al.*, 2012), which usually require <1 byte per input *k*-mer over the nucleotide alphabet {A,C,G,T}.

### 1.2 de Bruijn graph annotation

A major limitation of the above representations is that the identities of any sequence labels contained in the input text set are lost. To alleviate this, the concept of colored de Bruijn graphs emerged (Iqbal *et al.*, 2012) (otherwise known as annotated or labeled de Bruijn graphs), allowing for the representation of additional annotations per *k*-mer. These annotations can either be stored in conjunction with the *k*-mers or be organized in a separate data structure, using the *k*-mer representation only as an index space. Although the first option is used by a number of conceptually interesting methods, such as Mantis (Pandey *et al.*, 2018) that uses counting quotient filters to represent the *k*-mers linked to an annotation identifier, here we will only focus on the second option, as it allows for the connection of arbitrary annotations to the *k*-mer set, without re-processing the *k*-mer index.

Conceptually, the set of annotations is a relation between *k*-mers and labels that can be represented as a binary matrix, where the *k*-mer set indexes the rows and each annotation label specifies a column. Any entry (*i*, *j*) in the matrix represents the relation of *k*-mer *i* and annotation *j*. Different methods have been suggested to compress this annotation matrix in a way that still allows for efficient query. VARI (Muggli *et al.*, 2017, 2019) concatenates the rows of the annotation matrix and compresses the result using either an RRR (Raman *et al.*, 2007) or Elias–Fano coding (Elias, 1974; Fano, 1971).

Rainbowfish (Almodaresi *et al.*, 2017) takes advantage of high redundancy in matrix rows by computing a frequency code for the unique rows, compressing the unique rows in a matrix ordered by these codes and then representing the original matrix as a variable-length code vector. However, this method and other frequency coding-based approaches become less effective for datasets with greater levels of noise or inter-sample variability. Multi-BRWT (Karasikov *et al.*, 2020b) compresses the matrix in a hierarchical tree structure exploiting column similarity, but leaving the possible row redundancy unexploited. Alongside these methods, there is a rich literature of different compressors for graph annotations developed over the years, each improving on the compression performance of previous methods (Bingmann *et al.*, 2019; Harris and Medvedev, 2020; Marchet *et al.*, 2021). All of these methods share the common property that they act as general purpose binary matrix compressors, and thus, they do not take into account any particular domain knowledge in their construction.

### 1.3 Leveraging graph topology to improve annotation compression

While the methods mentioned above rely solely on similarities between annotation matrix elements to achieve their compression, a few have additionally leveraged graph topology to increase their compression potential. The Bloom filter correction method introduced by (Mustafa *et al.*, 2019) encodes the columns of the annotation matrix in Bloom filters with high false positive rate. Assuming that all vertices within a graph unitig (a path in which all vertices except for the first and last have in- and out-degree 1) share identical annotations, a row in the annotation matrix (corresponding to all vertices from the same unitig in the graph) is computed as the bit-wise AND of the rows stored for every vertex of that unitig. While achieving high accuracy in decoding row annotations, the corrected Bloom filters are not able to losslessly decode the rows of the encoded annotation matrix. In addition, the authors introduce a lossless approach based on wavelet tries which leverages graph backbone paths to improve compression performance. However, these paths must be provided by the user and cannot be computed automatically by the method.

The more recently introduced Mantis-MST method (Almodaresi *et al.*, 2019) constructs an annotation graph with nodes representing the unique rows of the annotation matrix. In this annotation graph, a weighted edge between two nodes *v*_1_ and *v*_2_ is created if there exist adjacent vertices *s*_1_ and *s*_2_ in the underlying de Bruijn graph whose annotations are represented by *v*_1_ and *v*_2_, respectively. The weight of this edge (*v*_1_, *v*_2_) is then set to the Hamming distance of the unique rows *v*_1_ and *v*_2_. Mantis-MST computes the minimal spanning tree of the annotation graph and represents the annotation of a node as it is bit-wise XOR with the annotation of its parent node in the spanning tree, while only the annotation of the root node is represented explicitly.

### 1.4 Our contribution

Despite the wide range of approaches summarized above that address the label-compression-problem for colored de Bruijn graphs, further improvements are needed to allow for applications in a multi-cohort, multi-metagenome (Tully *et al.*, 2018) or population-scale context (Bradley *et al.*, 2019). A key factor for reducing representation size is to leverage local similarities arising from evolutionary relationships of the input sequences. We present a new scheme for representing graph annotations, RowDiff, which takes advantage of similarities between the annotations of neighboring vertices to compress annotation matrices. RowDiff can be constructed using |G|+3m+O(|c|) bits of memory, where |c| is the compressed size of the largest column in the annotation matrix and |G| is the size of the memory representation of the graph and in this case is <4m+o(m) bits (Bowe *et al.*, 2012), where *m* is the number of *k*-mers, thus making it suitable for annotating virtually arbitrarily large graphs. Since RowDiff is a transformation of the input annotation matrix attempting to increase its sparsity, RowDiff can be naturally chained with any generic scheme for compressed binary matrix representation to achieve further improvements in compression performance. We demonstrate the compression performance of RowDiff relative to the state-of-the-art lossless Rainbow-MST and multi-BRWT methods on datasets representing different annotation matrix densities.

In the next sections, we define the underlying concepts (Sections 2.1 and 2.2) and detail this methods for construction (Sections 2.3 to 2.4) and querying (Section 2.5) of the RowDiff data structures. We then describe the test datasets (Section 3.1) and study the representation sizes (Sections 3.2, 3.3 and 3.4), construction time (Section 3.5) and query time (Section 3.6) of RowDiff-compressed annotations. Finally, we discuss limitations and directions for future work (Section 4).

## 2 Materials and method

### 2.1 Notation

We will operate in the following setting. Let *k* be a positive integer. The order *k* de Bruijn graph over a set of sequences *S*, denoted by $\mathrm{DB}G_{k}(S)$, is a directed graph $\mathrm{DB}G_{k}(S):=(V_{k},E_{k})$, whose vertices *V_k_* are the set of all distinct sub-strings of length *k* of sequences in *S* (*k*-mers), and an arc links $u\in V_{k}$ to $v\in V_{k}$, if $u_{2:k}=v_{1:k-1}$, where $s_{i:j}$ denotes the sub-sequence of *s* from position *i* up to and including position *j*. We denote with $\deg^{-}(v)$ and $\deg^{+}(v),v\in V_{k}$ the in- and out-degree of a vertex, respectively. Vertices $v\in V_{k},\deg^{-}(v)=0$ are called *source vertices* and vertices $v\in V_{k},\deg^{+}(v)=0$ are called *sink vertices*.

Given an arbitrary set of labels *L*, an *annotation* for a de Bruijn graph $\mathrm{DB}G_{k}(S)$ is a relation $A\subset V_{k}\times L$, which assigns to each vertex $v\in V_{k}$ a set of labels, l(v)⊂L. We will trivially represent A using a binary matrix $A\in {\left\{0,1\right\}}^{\left|V_{k}|\times |L\right|}$, denote with *A_i_* the *i*-th row of *A* and with $A_{i}⊕A_{j}$ the element-wise XOR of rows *i* and *j*.

### 2.2 RowDiff-transformation

RowDiff relies on the observation that adjacent vertices in the graph are likely similarly annotated, and thus, their respective rows in the annotation matrix *A* are similar as well. This implies that if $(u,v)\in E_{k}$, storing the difference between *A_u_* and *A_v_* may be more space efficient than storing *A_u_*, i.e. $\text{popcount}(A_{u}⊕A_{v})<\text{popcount}(A_{u})$, where popcount(x) represents the number of set bits in row *x*.

RowDiff is defined as a transformation that converts an annotation matrix *A* of a de Bruijn graph into a new, sparser, annotation matrix $A^{*}$ of the same size and an additional anchor vector $a\in {\left\{0,1\right\}}^{\left|V_{k}\right|}$, that is, ${\text{rd}}_{\mathrm{DB}G_{k}(S)}:\;{\left\{0,1\right\}}^{\left|V_{k}|\times |L\right|}\to {\left\{0,1\right\}}^{\left|V_{k}|\times (|L|+1\right)}$. The anchor vector ***a*** stores which rows remain unchanged. We show that the original annotation matrix *A* can be reconstructed from the RowDiff-transformed matrix $A^{*}$ and the anchor vector ***a***. Empirically, the RowDiff-transformed matrix is significantly better compressible in the typical case where neighboring vertices have similar annotations. We develop an efficient algorithm for defining anchors and for computing the RowDiff-transform ${\text{rd}}_{\mathrm{DB}G_{k}(S)}$ and its inverse.

For each vertex $u\in V_{k}$ we arbitrarily define its RowDiff successor as its lexicographically largest outgoing vertex succ(u), such that $(u,\text{succ}(u))\in E_{k}$ and $\text{succ}(u)\geq v\;\forall (u,v)\in E_{k}$, if such *u* exists. RowDiff replaces each row *A_u_* with the (likely sparser) delta relative to its RowDiff successor. For binary rows, the delta is simply the element-wise XOR, $A_{u}^{*}:=A_{u}⊕A_{\text{succ}(u)}$, while for non-binary rows, the delta could store the difference between the row and its successor. In this work, we focus on binary matrices. The previous equation implies that $A_{u}=A_{u}^{*}⊕A_{\text{succ}(u)}$, which gives us a simple formula for recursively reconstructing the original row. In order to be able to reconstruct the original annotation *A* from $A^{*}$, some rows are left unchanged. A vertex $v\in V_{k}$ for which the annotation is stored unchanged is called an *anchor* and its corresponding value in the anchor bit vector will be set to 1, $a_{v}=1$. Sink vertices do not have a RowDiff successor, and must thus be anchors.

Algorithm 1 shows the implementation of the *inverse* transformation ${\text{rd}}_{\mathrm{DB}G_{k}(S)}^{-1}$, which reconstructs the original row *A_i_* from the RowDiff representation $A^{*}$.

**Algorithm 1** Row annotation reconstruction

1. **function** ReconstructAnnotation(i)
2.   row $\leftarrow A_{i}^{*}$
3.   **while**  $a_{i}$ = 0 **do**  ▹ current vertex is not an anchor
4.     i ← succ(i)
5.     row ← row ⊕ $A_{i}^{*}$
6.   **end while**
7.   **return** row
8. **end function**

Starting from any vertex in the de Bruijn graph, Algorithm 1 defines a traversal leading to an anchor vertex, for which the annotation was not transformed. Since de Bruijn graphs may have cycles, additional anchor vertices might have to be assigned in order to break RowDiff cycles (those cycles where *every* vertex is a RowDiff successor relative to its predecessor in the cycle).

Proposition 1. *Algorithm 1 finishes for every starting vertex, if and only if every sink vertex in the graph is an anchor and every RowDiff cycle contains at least one anchor vertex.*

Proof. Assume the algorithm does not finish for a starting vertex *i*. This implies that $a_{\mathrm{suc}c^{k}(i)}=0,\forall k\in N$, where *succ^k^* is the *k*-th iterate of *succ*. Since the number of vertices in the graph is finite, there must exist l,m∈N,l≠m, s.t. $\mathrm{suc}c^{l}(i)=\mathrm{suc}c^{m}(i)$. Thus, $\left({\text{succ}}^{l}(i),{\text{succ}}^{l+1}(i),\ldots ,{\text{succ}}^{m}(i)\right)$ is a cycle and must therefore contain at least one anchor vertex, which contradicts the initial assumption. Proof of necessity is equally trivial. □

Proposition 2. *Algorithm 1 correctly reconstructs the original annotation row A_i_ for every vertex* $i\in V_{k}$.

Proof. The algorithm computes $A_{i}^{*}⊕A_{\text{succ}(i)}^{*}⊕\cdots ⊕A_{{\text{succ}}^{p}(i)}^{*}$, where $a_{{\text{succ}}^{p}(i)}=1$ and thus, $A_{{\text{succ}}^{p}(i)}^{*}=A_{{\text{succ}}^{p}(i)}$. By repeatedly reducing the last 2 terms using $A_{{\text{succ}}^{p-1}(i)}^{*}⊕A_{{\text{succ}}^{p}(i)}=A_{{\text{succ}}^{p-1}(i)}$, the original equation is reduced to *A_i_*, which is the desired value. □

Once the set of anchor vertices ***a*** satisfies Proposition 1, the RowDiff-transformed matrix $A^{*}$ together with the anchor indicator bitmap ***a*** encode the original annotation matrix.

### 2.3 Anchor assignment

In addition to the small set of anchors described in Proposition 1, we seek to cap the maximum RowDiff path length (i.e. a path taken by Algorithm 1) to a certain value *M* (typically between 10 and 100) by ensuring that at least every *M*-th vertex in a RowDiff path is an anchor, as described below. This guarantees that the number of iterations in Algorithm 1 is bounded by a constant, and thus the average time complexity of reconstructing a single row is O(l·M), where l≪|L| is the average number of set bits (labels) per row. At the same time, since anchor vertices require storing the original, less sparse annotation row, it is desirable to minimize the total number of anchor vertices in order to keep the popcount (and thus the compressed size) of the RowDiff annotation $A^{*}$ small.

**Fig. 1.**
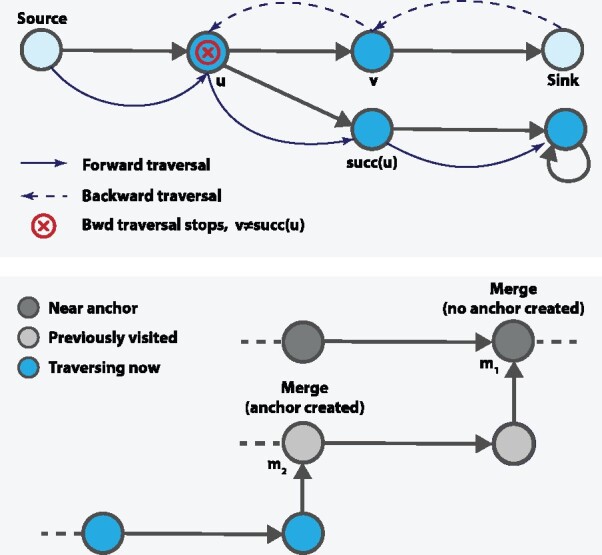
Top: RowDiff traversal. When traversing backward to assign anchor vertices, the traversal stops at vertex *u*, because succ(u)≠v. When traversing forward, the last outgoing vertex is selected. **Bottom:** Chained merge. Dark gray vertices are marked as nearAnchor. When traversing the light gray vertices, we merge into *m*_1_, marked as nearAnchor, thus, no anchor is set. When traversing the blue vertices, an anchor must be set at *m*_2_, as *m*_2_ is not marked as nearAnchor

The following anchor assignment algorithm allocates anchor vertices near-optimally in four steps as follows (Algorithm 2). First, we traverse RowDiff paths backwards (in parallel) starting from *sink vertices* (Supplementary Algorithm S1). The backward traversal stops either when we reach a source vertex or when we reach a vertex $v\in V_{k}$, s.t. succ(v)≠u for the previously traversed vertex *u* (Fig. 1, top). Note, the traversal is not terminated when reaching a vertex with multiple incoming arcs, but explores each of them and continues to further traverse these RowDiff paths backwards. When the distance from the current vertex to the next assigned anchor in the current RowDiff path reaches *M*, the vertex is marked as an anchor. In practice, once the backward traversal is finished, the vast majority of the vertices has been traversed, and the anchor assignment is optimal, in the sense that no anchors are closer than *M* to each other. In the second step, we start at *source vertices* and traverse RowDiff paths forwards, i.e. paths of the form $v,\text{succ}(v),{\text{succ}}^{2}(v),\ldots$ (Supplementary Algorithm S2). The traversal stops when we reach an already visited vertex. In the third step, we start traversing forward at all forks with unvisited vertices. After the third step, the only vertices that were not traversed must belong to a simple cycle (a cycle where all vertices have $\deg^{-}(v)=\deg^{+}(v)=1$). The fourth step traverses these cycles (in parallel). Each of these traversals sets an anchor every *M* vertices during the traversal. Since we visit each vertex only once, the time complexity of anchor assignment is $O(|V_{k}|)$.

**Algorithm 2** Anchor assignment

1. **function** AssignAnchors(*M*)
2.   visited[] ← {False} ▹ initialize mask of visited vertices
3.   anchor[] ← {False} ▹ initialize mask of anchor vertices
4.   **for all**  s∈Sinks()  **parallel do**
5.     anchor ← TraverseBwd(*s*, visited, anchor, *M*)
6.   **for all**  s∈Sources()  **parallel do**
7.     anchor ← TraverseFwd(*s*, visited, anchor, *M*)
8.   **for all**  s∈Forks()  **parallel do**
9.     anchor ← TraverseFwd(*s*, visited, anchor, *M*)
10.   ▹ only vertices in simple cycles (no forks) left unvisited at this point
11.   **for all**  s∈Nodes()  **parallel do**
12.     anchor ← TraverseFwd(*s*, visited, anchor, *M*)
13.   **return** anchor
14. **end function**

Proposition 3. *The anchors assigned by Algorithm 2 guarantee successful termination of Algorithm 1 for any input vertex* $v\in V_{k}$.

Proof. Step 1 of the algorithm trivially guarantees that all sink vertices are anchor vertices. Steps 3 and 4 guarantee that all RowDiff cycles in the graph are traversed and at least one anchor vertex is set in each cycle. The conditions in Proposition 1 are thereby satisfied and Algorithm 1 finishes and successfully reconstructs *A* from $A^{*}$.

One important detail in the forward traversal step is handling the situation when the traversal stops due to merging into a visited vertex. Not setting an anchor in such cases may result in arbitrarily long paths with no anchors (when such merges are chained). Always setting an anchor at a merge will introduce unnecessary anchors and increase the annotation density. We handle merges with the following simple heuristic: use an additional bit vector, nearAnchor, to mark all vertices that are known to be at a distance smaller than *M* to an anchor vertex. During forward traversal, when hitting a visited merge vertex that is marked in nearAnchor, no anchor is set (Fig. 1, bottom). A more optimal algorithm for deciding if a merge vertex should create an anchor would require labeling each vertex with the distance to its nearest anchor. In this implementation, we preferred the heuristic algorithm due to its significantly reduced space complexity.

#### 2.3.1 Anchor optimization

To guarantee that none of the rows $A_{v}^{*}$ in $A^{*}$ have more set bits than the corresponding row *A_v_* in the original annotation, we perform the following anchor optimization procedure. For each $v\in V_{k}$, s.t. $\text{popcount}(A_{v}^{*})>\text{popcount}(A_{v})$, we make such vertex an anchor, $a_{v}:=1$ and replace $A_{v}^{*}$ with *A_v_*. This ensures that all rows in the RowDiff-transformed annotation matrix are at least as sparse as the corresponding rows in the original annotation matrix.

Proposition 4*. Each row in a RowDiff-transformed annotation matrix has the same or fewer set bits than its corresponding row in the original annotation matrix.*

The anchor optimization procedure is implemented similarly to the initial construction of RowDiff (Section 2.4). Thus, it has the same time and space complexity.

### 2.4 Rowdiff construction

A naïve implementation of the RowDiff construction would be to load the matrix *A* in memory, and gradually replace its rows with their sparsified counterpart, while traversing the graph. Although fast and simple, this method requires to keep the entire annotation matrix *A* and the graph in memory. Unfortunately, this is often not realistic, as even the annotation matrix *A* alone can easily reach several terabytes in size. For this reason, we developed a distributed parallel construction algorithm that only loads a few columns of *A* at a time, and therefore needs only a limited amount of memory.

In the first stage, we load the graph and for each vertex pre-compute the indices of the unique RowDiff successor and the (possibly multiple) RowDiff predecessors, stored in vectors pred and succ, respectively. The pred and succ vectors are used to build $A^{*}$ in the second stage without the need to query the graph itself and load it in memory. To make the algorithm scale to de Bruijn graphs with trillions of vertices, vectors pred and succ are built and traversed in a streaming manner. They are loaded in small blocks, as described in Algorithm 3 and Supplementary Algorithm S3, and never kept in memory in full. Thus, pre-computing the pred and succ vectors essentially makes it possible to query the graph topology during the second stage while only using O(1) additional space. After the RowDiff annotation $A^{*}$ has been generated, the pred and succ vectors are not required for querying and, thus, can be discarded.

**Algorithm 3** RowDiff-transform

1. **function** Sparsify(columns)    ▹ sparsifies a batch loaded in memory
2.   **for**  block←0,numRows,BlockSize  **do**        ▹ Process by blocks
3.     **load** pred[block.block+BlockSize]
4.     **load** succ[block.block+BlockSize]
5.     **for all**  c∈columns  **parallel do**
6.       **for all**  i∈c[block..block+BlockSize]       **do**   ▹ Iterate only set bits
7.           **if not**  c[succ[i]]  **then**
8.             ▹ The bits at *i* and succ[i] are different, hence, diff ≠0
9.             $c^{*}[i]\leftarrow \mathrm{True}$
10.         **end if**
11.         **for all**  p∈pred[i]  **do**
12.           **if not**  c[p]  **then**
13.             ▹ The bits at *p* and *i* are different, hence, diff ≠0
14.             $c^{*}[p]\leftarrow \mathrm{True}$
15.         **end if**
16.       **end for**
17.     **end for**
18.   **end for**
19. **end function**

**Fig. 2.**
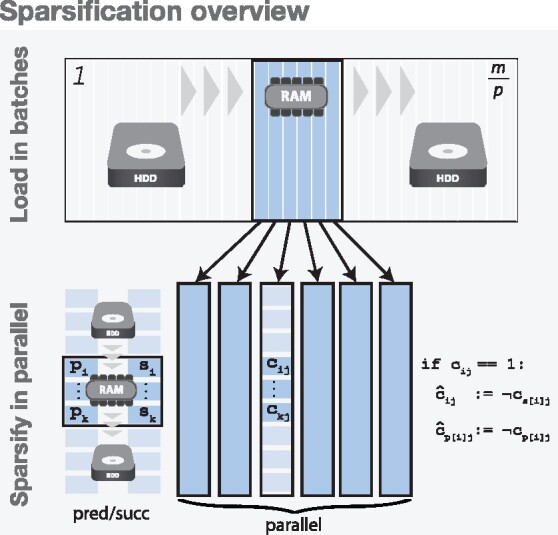
RowDiff-transform algorithm—Schematic overview of sparsification on a single machine. **Top:** Columns are loaded into memory in batches (until memory is exhausted) and each batch is fully transformed to RowDiff. The result is serialized and the process moves on to the next batch. **Bottom:** Each batch is transformed to RowDiff as follows. The algorithm iteratively loads into memory blocks of the pre-computed vectors pred and succ. Then, all columns of the batch are processed in parallel. The algorithm iterates only through set bits of each column in the active block and computes the elements of the RowDiff-transformed matrix $A^{*}$ (see Algorithm 3 for a more detailed description)

The second stage of the construction algorithm (the sparsification workflow) is schematically described in Figure 2. The initial sparsification of *A* can be trivially distributed by dividing the columns of *A* into groups and processing each group on a different machine. Each machine processes its assigned columns in batches. The size of each batch is determined dynamically by loading columns into memory until a desired upper limit is reached. This upper limit must be greater than the largest column being processed in compressed bit-vector format, but otherwise not restricted. For each column in the batch, we iterate only the set bits (only those rows corresponding to vertices annotated with the label represented by that column) and compare them with the bits at positions pred and succ in the same column to compute the RowDiff-transformed row, as shown in Figure 2.

#### 2.4.1 Scalability and complexity

Algorithm 3 only traverses set bits in *A*, and for each set bit in row *i* it performs $O(\deg^{-}(i)+1)$ operations, hence the total time complexity is O((1+α)popcount(A)), where *α* is the average in-degree of the graph. For de Bruijn graphs, α≤|Σ|, and hence the time complexity is linear in the number of set bits of the original annotation matrix, i.e. O(popcount(A)). Supplementary Algorithm S3, for constructing pred and succ, traverses each vertex exactly once, hence its time complexity is $O(|V_{k}|)$. Since the buffer used by Supplementary Algorithm S3 has a constant size, the space complexity is $\left|\mathrm{DB}G_{k}(S)|+O(1\right)$, where $\left|\mathrm{DB}G_{k}(S)\right|$ denotes the memory footprint of the graph, which, for instance, in the case of the BOSS representation (Bowe *et al.*, 2012) typically does not exceed 4m+o(m) bits, where $m=|V_{k}|$. After taking into account Algorithm 2 for anchor assignment, which requires three additional bits per vertex to indicate anchors and the traversal state, and putting it all together, we get that the RowDiff-transform can be performed in $O(\text{popcount}(A)+|V_{k}|)$ time and in $\left|\mathrm{DB}G_{k}(S)|+3|V_{k}|+O(|c|\right)$ space, where |c| is the memory footprint of the largest (densest) column of *A* in a compressed bit-vector format. Note that the first term in each of the sums is usually the dominant.

In terms of temporary disk space, the succ array takes $|V_{k}|\left\lceil \;\text{log}\;|V_{k}|\right\rceil$ bits and the pred array has similar size (in practice smaller). In addition, the anchor optimization algorithm needs 32 bits per vertex to count the number of bits per row. Thus, RowDiff construction needs in total <20 bytes per vertex of temporary disk storage.

In conclusion, we mention again that RowDiff construction can be easily distributed on multiple machines with modest hardware requirements and run in parallel on each machine, which makes the method very attractive for practical use on very large datasets.

### 2.5 Querying annotations for paths

We now note that, when querying annotations for paths in the graph or sets of rows corresponding to vertices from a local neighborhood in the graph, Algorithm 1 leads to redundant reconstruction work, as many of the queried rows belong to the same RowDiff paths. To alleviate this, we perform the traversal first and pre-compute all RowDiff paths from the rows queried. Then, we query all diff rows in one batch and reconstruct annotations for each row from the query. This ensures that no arc in these paths is traversed more than once. Moreover, querying all rows in one batch often allows making the query of the underlying representation of the sparsified binary matrix faster by exploiting its potential intrinsic features (e.g. jointly querying *n* bits in *m* columns is more cache-efficient and faster than *n* queries of single bits in each of the *m* columns).

### 2.6 Implementation details

We implemented RowDiff as part of the MetaGraph framework (Karasikov *et al.*, 2020a). The code for reproducing results of the experiments is available at https://github.com/ratschlab/row_diff. For storing original columns of the annotation matrix as well as the indicator bitmap with anchor vertices, we used the SD vectors from the sdsl-lite library (Gog *et al.*, 2014) for compressed representation of bitmaps. For compression of the transformed annotation matrix, we used the multi-BRWT representation scheme proposed in (Karasikov *et al.*, 2020b), with its improved and scaled up implementation from MetaGraph.

## 3 Results and discussion

In this section, we evaluate the performance of the methods described above both in terms of their final representation sizes and their construction time. In addition, we also study the effect of the maximum RowDiff path length on the final RowDiff representation size of the compressed annotations. Finally, we evaluate the degree of compression that RowDiff provides on a per-column basis.

### 3.1 Datasets

We evaluated the compression performance of RowDiff on three datasets with different levels of sequence variability and thus graph density. This first dataset consists of all Fungi sequences from RefSeq release 97 (O’Leary *et al.*, 2016), with annotations derived from the taxonomic IDs of the sequences’ respective organisms. This second and third datasets are derived from the cohort of 10 000 publicly available human RNA-seq experiments used in (Almodaresi *et al.*, 2019). We constructed annotated de Bruijn graphs from the RNA-seq dataset in the same manner as in (Almodaresi *et al.*, 2019), using a *k* value of 23, albeit with two samples discarded due to their withdrawal from the Sequence Read Archive. We will refer to this dataset as RNA-seq (*k* = 23). The third dataset is constructed using the graph cleaning approach originally developed by Turner *et al.* (2018) and scaled up in MetaGraph (Karasikov *et al.*, 2020a), using a *k* value of 31. We will refer to this dataset as RNA-seq (*k* = 31). For evaluating construction time and representation size, we shuffled the samples in each dataset and generated subsets of increasing size.

We evaluated RowDiff against MST (Almodaresi *et al.*, 2019), used in Mantis (Pandey *et al.*, 2018), which, to the best of our knowledge, is the most compact annotation representation method to date. Similarly to Rainbowfish (Almodaresi *et al.*, 2017), MST reduces the original annotation matrix to a set of unique rows and consists of two components: a vector, mapping indexes of rows of the annotation matrix to its unique rows (color classes) and the unique rows compressed in a minimum spanning tree. In Mantis, this mapping vector is included into a hash table storing the *k*-mers of the de Bruijn graph, which is usually at least an order of magnitude larger than the compressed annotation. Thus, to make a fair comparison, we exclude the large contribution of Mantis’ graph representation and only consider the mapping vector, using the same representation as in Rainbowfish (Almodaresi *et al.*, 2017). Thus, we refer to the MST annotation representation as Rainbow-MST. Note that Rainbow-MST forms a graph annotation representation which, similarly to RowDiff, can be used with any de Bruijn graph representation with indexed *k*-mers.

### 3.2 Representation size

We now compare the representation size for RowDiff and other state-of-the-art graph annotation compression methods. We additionally consider two binary matrix representation schemes: Multi-BRWT (Karasikov *et al.*, 2020b) and RowSparse for encoding the RowDiff-transformed annotation matrices. The RowSparse format stores the indices of set bits in each row in a compressed integer vector. This representation is faster to query than multi-BRWT, but its memory footprint is significantly larger.

**Fig. 3.**
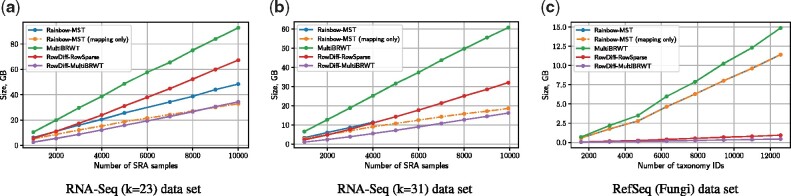
Representation size. The green line represents the size of the non-sparsified annotation matrix encoded with multi-BRWT. The purple and the red lines represent the size of the RowDiff annotation encoded with Multi-BRWT and RowSparse matrix representations, respectively. The blue line indicates the size of the Rainbow-MST annotation. The orange line represents the size of the Rainbow-MST mapping vector and represents a lower bound on the Rainbow-MST representation size. Rainbow-MST computation on the RNA-seq (*k* = 31) dataset with >4000 samples did not complete within the 10 days limit of this compute cluster. (**a**) RNA-seq (*k* = 23) dataset, (**b**) RNA-seq (*k* = 31) dataset and (**c**) RefSeq (Fungi) dataset

Figure 3 shows the representation size for the RNA-seq (*k* = 23), RNA-seq (*k* = 31) and RefSeq (Fungi) datasets. On the RNA-seq (*k* = 31) dataset, RowDiff-multi-BRWT effectively takes advantage of the topology of the graph annotation and the similarity of rows of the annotation matrix and achieves a nearly 4-fold size reduction compared to multi-BRWT applied on non-sparsified columns. Compared to the Rainbow-MST method, RowDiff-multi-BRWT achieves a 2-fold size reduction. Rainbow-MST computation on the subsets with more than 4000 samples could not be computed because Mantis did not complete within the 10 days limit of this compute cluster. For this reason, we also plotted the size of the Rainbowfish mapping vector, which, being a subset of the Rainbow-MST annotation data, represents a lower bound for Rainbow-MST.

On the RNA-seq (*k* = 23) dataset, RowDiff-multi-BRWT achieves a 2.5-fold size reduction relative to multi-BRWT and a 1.5-fold reduction relative to Rainbow-MST.

On the RefSeq (Fungi) dataset, RowDiff takes advantage of the longer stretches of vertices with identical annotations and achieves a 26-fold size reduction relative to Rainbow-MST (the detailed tabular data for this experiment are available in Supplementary Table S1). Notably, this significant difference is caused mainly by the large size of the mapping vector on this dataset.

### 3.3 Effects of graph density on compression

In this section, we analyze how the density of the annotated graph affects RowDiff compression. In a first experiment, we take a random subset of 1570 entries from the RefSeq (Fungi) dataset and build graphs and corresponding annotations for *k*-mer sizes ranging from 15 to 31. Table 1 shows how the compression ratio $\left|A|/|A^{*}\right|$ increases with higher sparsity of the graph (lower average out-degree for increasing *k*-mer length).

**Table 1.** Compression ratio versus graph density on a random subset of 1570 RefSeq (Fungi) annotation columns

*k*-mer size	Average out-degree	**Compression ratio** $\left|A|/|A^{*}\right|$
15	1.98	1.30
17	1.10	4.79
19	1.01	18.89
23	1.003	31.66
31	1.0017	34.53

*Note*: The sparser the graph the higher the compression ratio.

In the second experiment, we test how the maximum RowDiff path length *M* affects the annotation size for graphs of various densities. Table 2 shows the annotation size on the RNA-seq (*k* = 23), RNA-seq (*k* = 31) and RefSeq (Fungi) datasets for various values of *M*, where the column *M* = 0 corresponds to the size of the original annotation matrix without applying RowDiff. While increasing the maximum path length has negligible effect on the denser RNA-seq graphs (with average node degrees of 1.08 and 1.04, respectively), it reduces the annotation size by a factor of up to 5.7 (from 1.52 GB to 265 MB) on the much sparser RefSeq (Fungi) graph (with an average node degree of 1.003). This phenomenon can be explained by the observation that in sparser graphs the majority of the anchor nodes are set to restrict the maximum RowDiff path length, while in denser graphs the majority of the anchor nodes are set during the anchor optimization stage.

**Table 2.** Annotation size (in GB) versus maximum RowDiff path length *M* for RNA-seq (*k* = 23 and 31) and Refseq Fungi (*k* = 31)

Dataset	*M* = 0	*M* = 10	*M* = 25	*M* = 50	*M* = 75	*M* = 100
RNA-seq (*k* = 23)	214	125.1	119.8	118.3	118.0	117.8
RNA-seq (*k* = 31)	151	70.7	64.9	63.2	62.6	62.2
RefSeq (Fungi)	11.2	1.52	0.713	0.419	0.317	0.265

*Note*: Column *M* = 0 shows the size of the original columns not transformed with RowDiff.

### 3.4 Compression of single columns

**Fig. 4.**
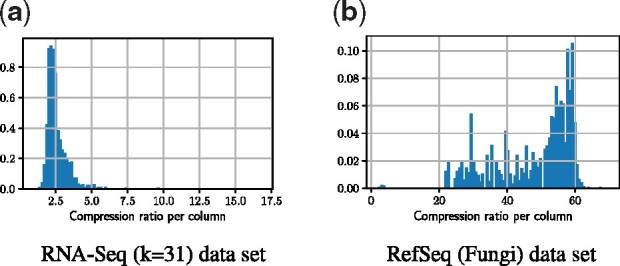
Histogram of the compression ratio per column $\left|A_{\cdot ,i}|/|A_{\cdot ,i}^{*}\right|$, weighted by the original column size. On the denser RNA-seq (*k* = 31) graph, the compression ratio peaks at around 2×, while on RefSeq (Fungi) the compression ratio peaks at ≈60×. The size of columns is measured by the memory footprint of their SD compressed representations. (**a**) RNA-seq (*k* = 31) dataset and (**b**) RefSeq (Fungi) dataset

In this experiment, we measure how RowDiff compresses individual columns of the annotation matrix. Figure 4 shows the compression ratio $\left|A_{\cdot ,i}|/|A_{\cdot ,i}^{*}\right|$ achieved by RowDiff on two datasets representing two different extreme cases of sequence variability.

The de Bruijn graph constructed from assembled genomes RefSeq (Fungi) contains significantly fewer branches and bubbles than the graph constructed from reads RNA-seq (*k* = 31), thus its annotation is significantly better compressed by RowDiff, with an average compression ratio of 42 (from 11.2 GB to 265 MB).

### 3.5 Construction time

**Fig. 5.**
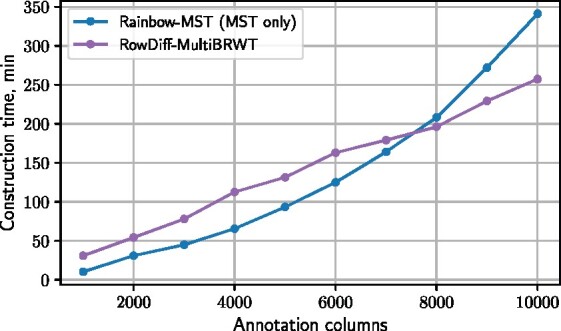
Construction time for the RowDiff and MST annotation representations on the RNA-seq (*k* = 23) dataset with 72 threads

In Figure 5, we compare the construction times for RowDiff and MST (Almodaresi *et al.*, 2019). The construction time for RowDiff-multi-BRWT includes everything from the RowDiff-transform of the original columns (with *M *=* *100) to the conversion of the transformed RowDiff columns to the multi-BRWT binary matrix representation. For MST, we only measure the time taken for compression of the unique annotation rows and do not include the time taken to construct the mapping vector (which is done by Mantis at the same time as constructing the graph and usually takes orders of magnitude longer to than construction of the MST part itself). Thus, this makes up a lower bound on the total construction time for the MST method. Note that the construction time for RowDiff-multi-BRWT grows linearly in the number of columns of the annotation matrix, and superlinearly for MST, which makes RowDiff a more favorable approach on a larger scale.

### 3.6 Query performance

In this experiment, we measured the time needed to query human transcripts against the RNA-seq (*k* = 23) annotation.

Table 3 shows the time taken to query 100 and 1000 random human transcripts for the baseline multi-BRWT matrix representation method, the Mantis-MST method and the proposed RowDiff method (with the RowDiff matrix encoded using multi-BRWT or RowSparse).

**Table 3.** Time for querying 100 and 1000 random human transcripts with multi-BRWT, Mantis-MST and RowDiff methods

Query data	**# rows** **queried**	Query time			
		Multi	Mantis	RowDiff	RowDiff
		BRWT (s)	MST (s)	RowSparse (s)	Multi-BRWT (s)
100 trans.	44 995	51	4.5	8.3	40
1000 trans.	553 280	226	68	54	197

*Note*: The second column shows the total number of annotation rows queried. All benchmarks were performed with a single thread on Intel(R) Xeon(R) Gold 6140 CPU @ 2.30 GHz.

We queried RowDiff annotations using the algorithm optimized for long paths (Section 2.5). First, we construct a list of annotation rows that have to be reconstructed from the RowDiff format and a list of all diff rows for querying in the RowDiff matrix. Then, all these rows are queried at once and the original annotation rows are reconstructed. Since RowDiff additionally requires traversing the de Bruijn graph to get RowDiff paths, the query time for RowDiff depends on the traversal performance of the underlying representation of the de Bruijn graph. In this experiment, we used the succinct de Bruijn graph representation available in the MetaGraph library.

For Mantis-MST, the total time reported by Mantis was measured, excluding the loading time. This also includes the time taken to map the *k*-mers from the query to rows of the annotation matrix, which could not be subtracted because Mantis only reports the total query time. However, as querying the annotation matrix is the bottleneck of the query algorithm, this makes up a relatively small fraction of the total time.

**Fig. 6.**
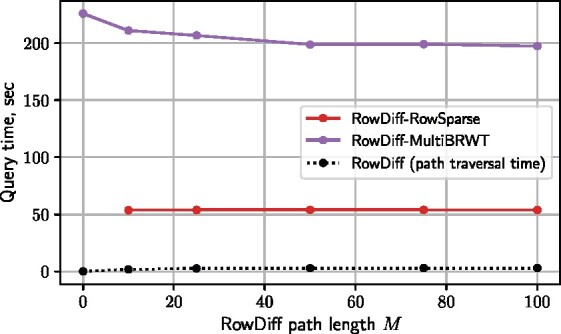
Query time for different values of the maximum RowDiff path length *M*. The first point *M *=* *0 corresponds to the baseline multi-BRWT encoding the original annotation matrix without RowDiff

Finally, we study the influence of the maximum RowDiff path length parameter *M* on the query performance of RowDiff (Fig. 6 and Supplementary Figs. S1 and S2). Notably, setting a larger value of *M* not only increases the compression ratio but also makes queries on RowDiff-multi-BRWT faster (in the range of studied values from *M *=* *10 to 100), which can be explained by the higher performance of multi-BRWT on sparser matrices. In contrast, RowDiff-RowSparse does not show any noticeable dependence of the query time on *M*. As mentioned above, the query time for RowDiff depends on the graph traversal speed because of the required traversal of RowDiff paths to their anchors. However, Figure 6 shows that the traversal actually takes a relatively small part of the total time. Hence, further improvements of RowDiff increasing the sparsity of the annotation matrix will likely make queries faster.

## 4 Conclusions

In this article, we introduced RowDiff, a new technique for compacting graph labels by leveraging the likely similarities in annotations of nodes adjacent in the graph. We designed a parallel construction algorithm with linear time complexity in the number of node-label pairs and small memory footprint. In addition, the algorithm can efficiently be distributed and parallelized, making it applicable virtually on arbitrarily large graphs. RowDiff reduced the size of graph annotations by 2- to 26-fold when used in combination with multi-BRWT relative to Mantis-MST, the smallest state-of-the-art representation.

Although the row reconstruction method inevitably leads to an increase in *ad hoc* row query time due to the larger number of required annotation matrix queries, this limitation is alleviated in practice due to the tendency of real-world sequences to feature *k*-mers which co-occur on matching RowDiff paths, which results in overall smaller query times.

The optimization of anchor assignment is a clear direction for future development of these methods. The anchor assignment method we have presented is designed to reduce the row reconstruction time by setting an upper bound on the traversal length. However, given that there is a trade-off between the size and the query time of the final representation, designing an objective function and a corresponding algorithm to best optimize these measures is a non-trivial task.

Moving beyond the representation of binary relations, a simple extension of the RowDiff method can be used as an efficient way to represent genomic coordinates for indexes of reference genomes. By representing a coordinate at each anchor node, the coordinates of all other nodes in that anchor’s corresponding RowDiff path can be computed via their traversal distance to the anchor.

Each improvement in the compression of sequence graphs and their associated annotations opens up further opportunities for their real-world applicability. When handling large annotations, even a 2-fold difference in the representation size can make a previously unapproachable annotation accessible to the available hardware. With RowDiff, we have demonstrated that there still is great potential for improving the representation of annotations on sequence graphs.

## Funding

This work was supported by the Swiss National Science Foundation [407540_167331 to G.R. for M.K. and H.M.] and ETH core funding to G.R. (for A.K. and D.D.).

*Conflict of Interest*: none declared.

## Supplementary Material

btab330_Supplementary_Data
