# Supplementary material for: Topology-based sparsification of graph annotations
Source: Bioinformatics. 2021 Jul 12;37(Suppl 1):i169–76. doi: 10.1093/bioinformatics/btab330 (PMC8346655; doi:10.1093/bioinformatics/btab330)
Supplement: btab330_Supplementary_Data [file btab330_supplementary_data.pdf]

Supplementary Material
Topology-based Sparsification of Graph Annotations

Daniel Danciu [1,2,*] Mikhail Karasikov [1,2,3,*] Harun Mustafa [1,2,3]
André Kahles [1,2,3,†] Gunnar Rätsch [1,2,3,4,†]

[1]Biomedical Informatics Group, Department of Computer Science, ETH Zurich, Zurich, Switzerland
[2]Biomedical Informatics Research, University Hospital Zurich, Zurich, Switzerland
[3]Swiss Institute of Bioinformatics, Zurich, Switzerland
[4]Department of Biology, ETH Zurich, Zurich, Switzerland

Algorithm S1 Backward traversal for anchor assignment

```
function TraverseBwd(sink, visited[], anchors[], M)
    anchor[sink] ← True                                ▷ mark the sink as anchor
    queue.push(sink, 0)                                ▷ distance to next anchor is zero
    while not queue.empty() do
        node, depth ← queue.pop()
        if not visited[node] then                      ▷ for detecting loops
            visited[node] ← True
            if depth = M then
                anchor[node] ← True
                depth ← 0
            end if
            if Last(node) then                         ▷ only for last outgoing nodes
                for all n ∈ Incoming(node) do
                    queue.push(n, depth+1)             ▷ go further from anchor
                end for
            end if
        end if
    end while
end function
```

*Joint-first authors.
†Joint corresponding authors; contact: andre.kahles@inf.ethz.ch and gunnar.ratsch@ratschlab.org.

Algorithm S2 Forward traversal for anchor assignment

```
function TraverseFwd(node, visited[], anchor[], nearAnchor[], M)
    path ← []
    while not visited[node] do                          ▷ Traverse until hitting a merge
        visited[node] ← True
        path.append(node)
        node ← lastOutgoing(node)
    end while
    for i = 0, len(path) − M, M do                      ▷ Assign anchor every M nodes
        anchor[path[i]] ← True
        nearAnchor[path[i:i+M]] ← True
    end for
    next ← lastOutgoing(path.back())
    if len(path) mod M == 0 or nearAnchor[next] then
        return                                          ▷ We merged close to an anchor, done
    end if
    anchor[path.back()] ← True
    nearAnchor[path[i:]] ← True
end function
```

Algorithm S3 Streaming construction of the `pred` and `succ` vectors

```
function CreatePredSucc
    for start = 0, |V|, BLOCK_SIZE do                   ▷ traverse the graph in blocks of size BLOCK_SIZE
        parfor i = start, start+BLOCK_SIZE do
            if a[i] = 0 then                            ▷ Anchors don't have a successor
                succBuf[threadNo].append(lastSucc(i))
            end if
            for all j in pred(i) do
                if succ(j)=i then                       ▷ Check if i is the RowDiff successor of j
                    predBuf[threadNo].append(j)
                    predBoundaryBuf.append(0)
                end if
            end for
            predBoundaryBuf.append(1)
        end parfor
                                                        ▷ the following commands dump the memory buffer to disk
        succ.append(succBuf), pred.append(predBuff), predBoundary.append(predBoundaryBuf)
    end for
end function
```

Table 1: **Representation size** of the RefSeq (Fungi) graph annotation and its subsets, in MB. The second column shows the memory footprint of the original annotation columns compressed in SD vectors.

# columns	Columns $\lvert A \rvert$	Multi-BRWT	RowDiff $\lvert A^* \rvert$	RowDiff with anchors	RowDiff-RowSparse	RowDiff-MultiBRWT	Rainbow-MST (mapping only)	Rainbow-MST (MST only)	Rainbow-MST
1570	664.5	692.8	14.4	22.8	61.0	25.2	559.4	0.2	559.5
3140	1834.1	2186.5	38.4	58.9	152.8	68.0	1753.9	0.6	1754.4
4710	2998.6	3502.5	62.8	93.1	234.7	108.6	2781.3	1.0	2782.4
6280	4630.6	5966.9	102.6	147.1	380.7	175.0	4629.9	2.0	4631.8
7850	6628.9	7873.8	147.7	208.2	527.5	246.9	6282.2	2.9	6285.2
9420	8224.9	10248.4	187.4	260.3	666.7	312.7	8013.5	4.2	8017.6
10990	9640.5	12320.8	224.7	310.0	790.0	371.7	9613.9	5.4	9619.3
12561	11214.5	14879.1	265.3	361.3	928.5	430.0	11409.5	7.0	11416.5

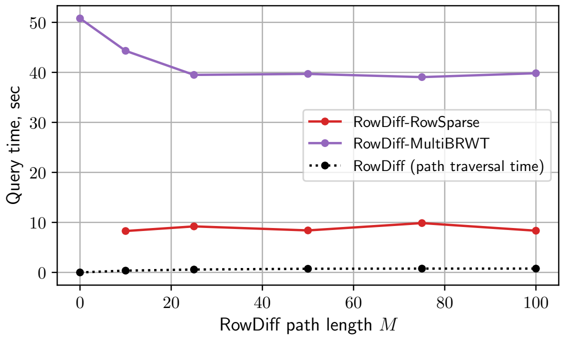

(a) Query time summary

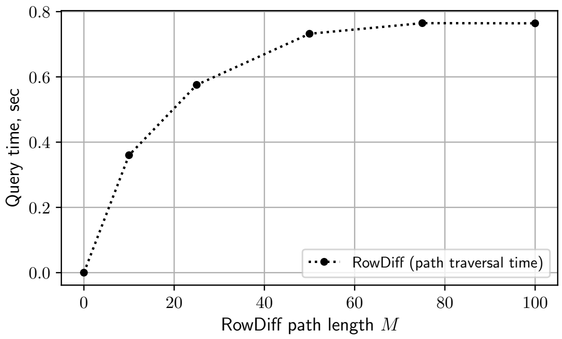

(b) Only RowDiff path traversal time

Figure 1: **Query time** for RowDiff on 100 random human transcripts.

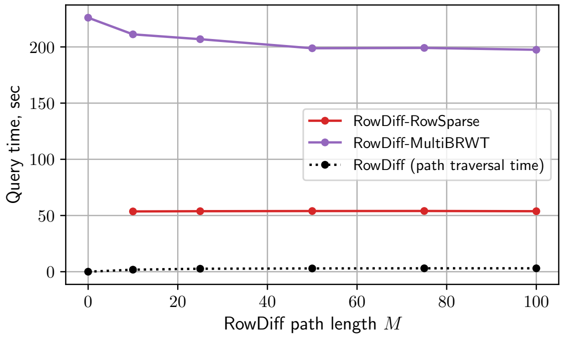

(a) Query time summary

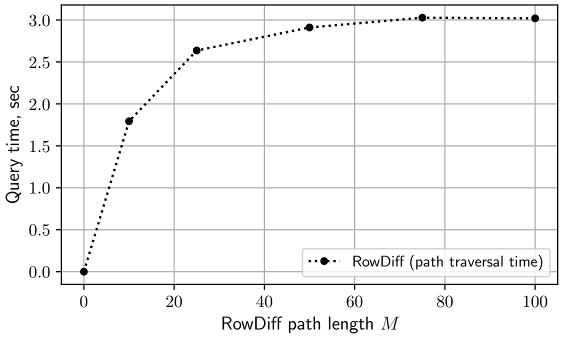

(b) Only RowDiff path traversal time

Figure 2: **Query time** for RowDiff on 1000 random human transcripts.
